# Development and validation of the MultiScent-20 digital odour identification test using item response theory

**DOI:** 10.1038/s41598-024-65915-3

**Published:** 2024-07-01

**Authors:** Marcio Nakanishi, Pedro Renato de Paula Brandão, Gustavo Subtil Magalhães Freire, Luis Gustavo do Amaral Vinha, Marco Aurélio Fornazieri, Wilma Terezinha Anselmo-Lima, Danilo Assis Pereira, Gustavo Henrique Campos de Sousa, Claudia Galvão, Thomas Hummel

**Affiliations:** 1 Department of Otorhinolaryngology, University Hospital of Brasília, Graduate Program in Medical Sciences, School of Medicine, University of Brasília, Brasília, DF Brazil; 2 Research Institute, Hospital Sírio-Libanês, Brasília, DF Brazil; 3 School of Medicine, University of Brasília, Brasília, DF Brazil; 4 Statistics Department, University of Brasília, Brasília, DF Brazil; 5 State University of Londrina, Pontifical Catholic University of Paraná, Londrina, Brazil; 6 Department of Ophthalmology and Otorhinolaryngology, Ribeirão Preto Medical School-University of São Paulo, Ribeirão Preto, Brazil; 7 Brazilian Institute of Neuropsychology and Cognitive Sciences, Brasília, DF Brazil; 8 NOAR Brasil Ltda, São Paulo, SP Brazil; 9 Department of Otorhinolaryngology, Smell and Taste Clinic, Technische Universität Dresden, Dresden, Germany

**Keywords:** Psychology, Biomarkers, Neurological disorders, Respiratory tract diseases, Olfactory system, Biomedical engineering, Olfactory system, Neuroscience, Sensory processing

## Abstract

Although validated and reliable psychophysical tests of olfactory function are available, an easy-to-use and feasible test has yet to be developed. This study aimed to design a digital odour identification test, evaluate its validity, assess its reliability, establish a normative curve, and explore the impact of demographic factors. The odour identification test was presented with the Multiscent-20, a hand-held, tablet-like digital scent device that features an integrated odour digital delivery system. The identification performance on the 20 odours was assessed using item response theory (IRT). The normative curve was established by administering the test to a large sample of participants (n = 1299). The mean identification score was 17.5 (SD = 2.1). The two-parameter logistic IRT model provided the best fit, revealing variation in item discrimination and difficulty parameters. Educational attainment influenced performance, with primary education associated with lower scores. Additionally, sex was not found to be associated with performance. This study provides initial evidence supporting the validity and reliability of use of the Multiscent-20 as a digital odour identification test. The test’s automation and portability enable the standardized delivery of olfactory stimuli and efficient automatic recording and scoring of responses.

## Introduction

Olfactory function assessment is vital for evaluating human health, providing information about an individual’s neurological and cognitive status. At present, there are reliable and validated psychophysical tests for olfactory function assessment in clinical practice, including the University of Pennsylvania Smell Identification Test (UPSIT)^1^, which employs microencapsulation of odorous liquids, and the Sniffin’ Sticks test^2–4^, which uses felt-tip pens infused with odorized fluids. While these tests are elegant and widely used, they have some limitations, such as the time required for testing, repeated use, cost, and the requirement of having a trained examiner conduct the tests. As a result, there is an increasing need for automated testing procedures capable of delivering stimuli, recording responses, and scoring assessments in a cost-effective manner. To overcome these challenges, we developed the Multiscent-20 (Noar, São Paulo, Brazil), a portable tablet computer with an integrated odour dispensing system; this device provides an efficient, mobile, and user-friendly method for olfactory function assessment.

A recent proof-of-concept study demonstrated the feasibility of using the Multiscent-20 to present an olfactory function assessment^5^. We enlisted 180 participants and compared performance on the Multiscent-20 with that on the 40-item UPSIT. The findings indicated that participants completed this test in a shorter duration and that there was a strong correlation between performance on the two tests. The Multiscent-20 showed high test–retest reliability and was regarded as easy to use. Additionally, due to the COVID-19 pandemic, recent years have seen a surge in interest in olfactory function evaluation and research, underscoring the need for a universally accepted, feasible assessment tool that is validated across diverse populations globally^6,7^. The creation of such a tool would substantially expedite cross-cultural research and ensure consistent evaluation criteria among diverse populations. To address this demand, we endeavoured to develop a new self-administered digital olfactory function test based on the classic 4-alternative forced-choice (AFC) paradigm to provide a universally applicable olfactory function assessment.

In this study, we describe the development, validation, and establishment of the normative curve of the Multiscent-20 olfactory identification test in its preliminary version. The selection of odours for this digital assessment was predicated upon the assumption that they embodied universally recognized and familiar odours across all five continents, thereby ensuring the potential to apply this assessment in a variety of cultures^6^.

Evaluating the reliability and identification performance on individual items in any psychophysical test is crucial to ensure the test’s overall validity. Item response theory (IRT) is a framework for examining these elements, modelling the relationship between an individual’s latent trait (olfactory ability, in this instance) and their responses to individual test items^8^. IRT enables assessment of the interplay between item parameters, such as difficulty and discrimination, in conjunction with the latent ability of tested individuals, thereby enhancing the precision of measurement and facilitating adjustments and adaptations for diverse test versions.

The objectives of this study are as follows:

- To develop an odour identification test utilizing the Multiscent-20 that can effectively and adequately measure individual olfactory identification ability.
- To assess performance on the 20 items of the Multiscent-20 through IRT analysis, ensuring accuracy and reliability, and examining item parameters such as discrimination and difficulty. Additionally, this assessment will gauge the quality of information generated by the test.
- To establish the normative curve and investigate the influence of demographic factors on participant performance.

## Results

### Participants

Table 1 displays the demographic and clinical data of a sample of 1832 individuals. The majority of the participants reported that they did not have a cold or flu at the time of testing (85%), difficulty smelling things (90%), or major memory issues (98%). Similarly, most participants reported that they were literate (99%) and had never had a head injury (98%). Among those who underwent COVID-19 testing, 31% tested positive, and 19% of those who tested positive reported a loss of smell. The majority of participants had completed secondary education (69%), followed by those who had completed tertiary education (23%), and those who had completed only primary education (8%). Most participants were male (55%). The mean age of the sample was 37 years, with the male participants exhibiting a slightly higher mean age than the female participants (38.4 vs. 34.8 years, respectively).

**Table 1:** Demographic and clinical data of the study participants.

Characteristic	n	%
Sex (n = 1832)		
Male	1005	54.8
Female	827	45.2
Age group		
18–30 years	551	30.1
30–40 years	438	23.9
40–50 years	664	36.2
50–60 years	157	8.6
> 60 years	22	1.2
Educational attainment (n = 1832)		
Primary education	144	7.8
Secondary education (high school)	1260	69.1
Tertiary education (university)	428	23.1
Cold or flu-like symptoms (n = 1710)		
No	1447	84.6
Yes	243	14.2
No answer/not sure	20	1.2
Literate		
Yes	1690	98.8
No	16	1.0
No answer/not sure	4	0.2
Difficulty smelling things		
No	1543	90.2
Yes	135	7.9
No answer/not sure	32	1.9
Severe memory problems		
No	1665	97.4
Yes	34	2.0
No answer/not sure	11	0.6
Head injury with loss of consciousness		
No	1674	97.9
Yes	29	1.7
No answer/not sure	7	0.4
COVID-19 test result		
Negative	1161	67.9
Positive	531	31.1
No answer/not sure	18	1.0
Loss of sense of smell with COVID-19		
Yes	327	61.6
No	202	38.0
No answer/not sure	2	0.4
Regained sense of smell lost due to COVID-19		
Yes	266	15.5

### Dimensionality assessment

The unidimensionality of test results was evaluated through confirmatory factor analysis (CFA). The factor model fit statistics were robust. The chi-square test for the factor model yielded a significant result (χ^2^(170) = 245.189, p < 0.001), indicating a good fit. The model also demonstrated strong performance in various other fit indices, including the comparative fit index (CFI = 0.983), Tucker‒Lewis index (TLI = 0.981), and Bentler-Bonett normed fit index (NFI = 0.947). Moreover, the model exhibited a low root mean square error of approximation (RMSEA) value of 0.015, with a 90% confidence interval ranging from 0.011 to 0.020. Additionally, the standardized root mean square residual (SRMSR) was well within acceptable limits (0.067). In summary, the CFA provided strong support for unidimensionality of test results.

### Item-level IRT analysis

#### Selection of the most suitable IRT model

Three IRT models were compared to determine the best-fitting model for the data: the one-parameter logistic (1PL) model, the two-parameter logistic (2PL) model, and the three-parameter logistic (3PL) model. The goodness-of-fit statistics for each model included the M2, RMSEA, SRMR, TLI, and CFI values.

The 1PL model showed a relatively poor fit, with an M2 of 1046.848 (df = 189, p < 0.001), RMSEA of 0.0495, SRMSR of 0.0914, TLI of 0.8458, and CFI of 0.8466. In contrast, the 2PL model demonstrated a considerably better fit, with an M2 of 316.1901 (df = 170, p < 0.001), RMSEA of 0.0216, SRMSR of 0.0395, TLI of 0.9708, and CFI of 0.9739. The 3PL model showed a similarly good fit, with an M2 of 258.4386 (df = 150, p < 0.001), RMSEA of 0.0198, SRMSR of 0.0409, TLI of 0.9754, and CFI of 0.9806.

A comparison of the three models using the Akaike information criterion (AIC), Schwarz’s Bayesian information criterion (SABIC), Hannan-Quinn information criterion (HQ), and Bayesian information criterion (BIC) revealed that the 2PL model had the lowest values for all criteria, indicating the best fit to the data (Table 2). Furthermore, an analysis of variance (ANOVA) comparing the three models showed a significant improvement in fit for the 2PL model over the 1PL model (χ^2^ = 327.282, df = 19, p < 0.001) but no significant difference in fit between the 2PL and 3PL models (χ^2^ = 19.075, df = 20, p = 0.517).

**Table 2:** Comparative analysis of information criteria for item response theory models.

Model	AIC	SABIC	HQ	BIC	logLik	χ^2^	df	p-value
1PL	23,833.51	23,882.79	23,876.27	23,949.51	− 11,895.75	–	–	–
2PL	23,544.22	23,638.11	23,625.67	23,765.19	− 11,732.11	327.282	19	< 0.001
3PL	23,565.15	23,705.97	23,687.32	23,896.59	− 11,722.58	19.075	20	0.517

*AIC* Akaike Information Criterion, *SABIC* Schwarz’s Bayesian Information Criterion, *HQ* Hannan–Quinn information criterion, *BIC* Bayesian Information Criterion, *logLik* Log-likelihood of the model, *χ*^*2*^ Chi-squared statistic, *df* degrees of freedom.

Based on these results, the 2PL model was chosen as the most suitable model for the data. This model provides a more flexible approach than the 1PL model by allowing items to have different discrimination parameters, which can better capture the underlying relationships between the items and the latent trait. The lack of significant improvement in fit from the 2PL model to the 3PL model suggests that the added complexity of the 3PL model, which incorporates a guessing parameter, was unnecessary for adequate representation of the data.

#### Item and person fit

The item fit parameters for the 2PL IRT model were assessed using S-χ^2^, RMSEA, and p values. The S-χ^2^ statistic evaluates the discrepancy between observed and expected item responses, with higher values indicating a worse fit. RMSEA provides an estimate of the item fit error, with values closer to 0 indicating a better fit. The p value assesses the statistical significance of the item misfit.

In the present analysis, most items exhibited good fit, with nonsignificant p values (> 0.05) and low RMSEA values. However, a few items showed potential misfits: Item 3 (Coconut) with an S-χ^2^ of 26.757, df = 12, RMSEA = 0.026, and p value = 0.008; Item 6 (Tire) with an S-χ^2^ of 23.440, df = 12, RMSEA = 0.023, and p value = 0.024; and Item 14 (Strawberry) with an S-χ^2^ of 23.926, df = 11, RMSEA = 0.025, and p value = 0.013.

The person-fit statistics for the 2PL IRT model indicated that the majority of individual response patterns were consistent with the model expectations, with 99.946% and 98.920% fitting based on infit and outfit statistics, respectively.

#### Discrimination and difficulty parameters

The discrimination (a) and difficulty (b) parameters of the 2PL IRT model for the 20 items provided meaningful insights into the characteristics of the test items. The discrimination parameters ranged from 0.38 to 2.43, indicating that items vary in their ability to differentiate between individuals with differing levels of the underlying latent trait. For instance, the item ‘Vanilla’ (a = 2.43) showed higher discrimination, meaning that it better distinguished between individuals along the ability continuum. In contrast, the item ‘Rose’ (a = 0.38) had lower discrimination and was less effective in this regard (Supplementary Table S2).

**Figure 1:**
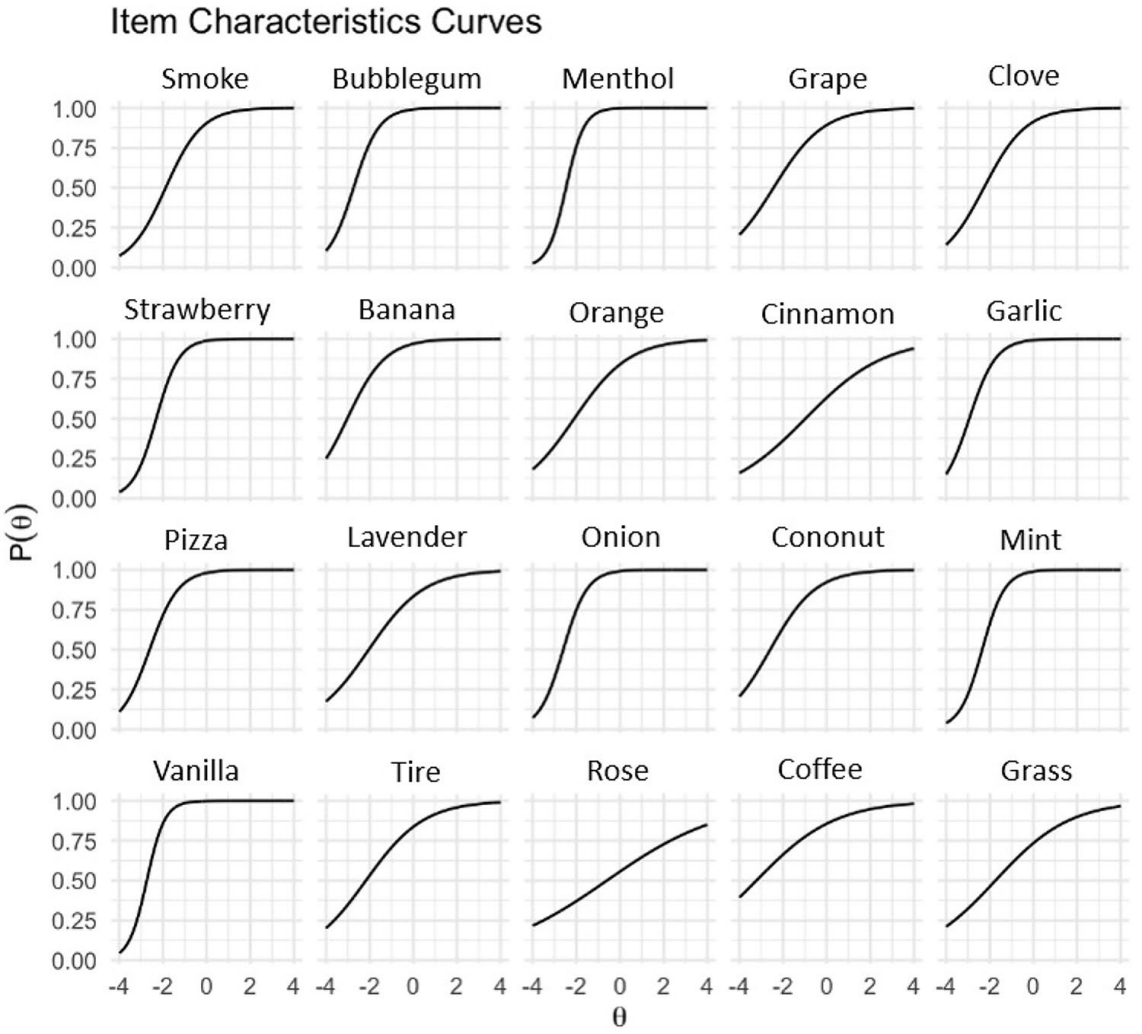
Item Characteristic Curves. Each curve depicts the relationship between an individual’s odour identification ability (θ, theta) on the horizontal axis and the probability of providing a correct answer to the question on the vertical axis. The person parameter (θ, latent trait, or ability) is on a scale from − 4 (severely impaired odour identification ability) to + 4 (excellent odour identification ability). The P(θ) depicted on the y-axis of each curve represents the probability of providing the correct answer on the specific item.

**Figure 2:**
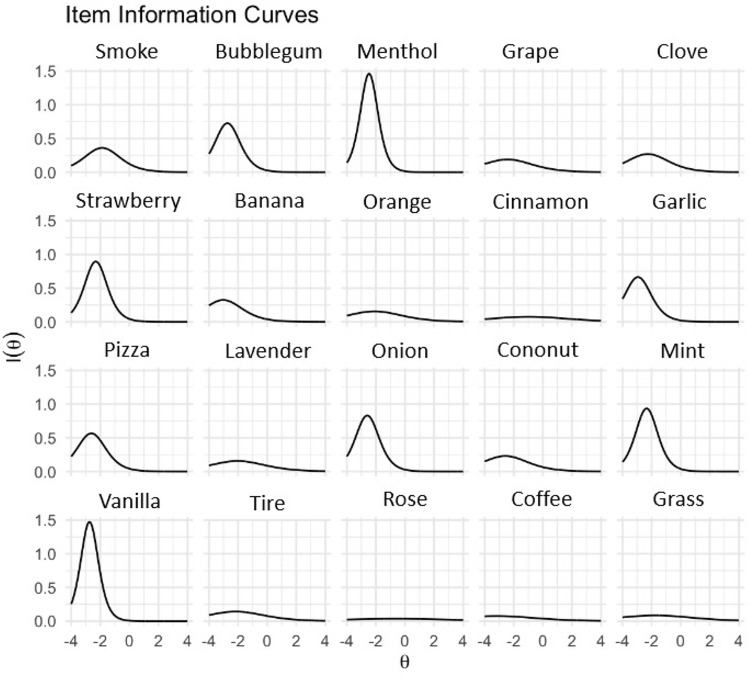
Item Information Curves. Each curve represents an item in the Multiscent-20. The shape of the curve indicates how much information the item provides at different levels of latent ability. A steeper curve indicates that the item offers more information at a given level of odour identification ability, while a flatter curve indicates that the item provides less information. The person parameter (θ, latent trait, or ability) is on a scale that goes from − 4 (low odour identification ability) to + 4 (high odour identification ability). The curves show that Menthol, Bubble-gum, Strawberry, Garlic, Onion, Pizza, Mint, and Vanilla are the most informative items.

Figure 1 presents the item characteristic curves (ICCs) for the Multiscent-20 items, illustrating the relationship between individual ability and item difficulty on the same dimension. Figure 2 shows the item information curves (IICs), visually depicting the contribution of individual items to the overall precision of the scale.

The difficulty parameters ranged from − 3.22 to − 0.60, providing information about the relative difficulty of each item. A more negative value of difficulty corresponds to an easier item, while a less negative value of difficulty denotes a more difficult item. For example, the item ‘Coffee’ (b =  − 3.22) is relatively easier to identify, whereas the item ‘Rose’ (b =  − 0.60) is more challenging for test-takers.

### Test information curves

**Figure 3:**
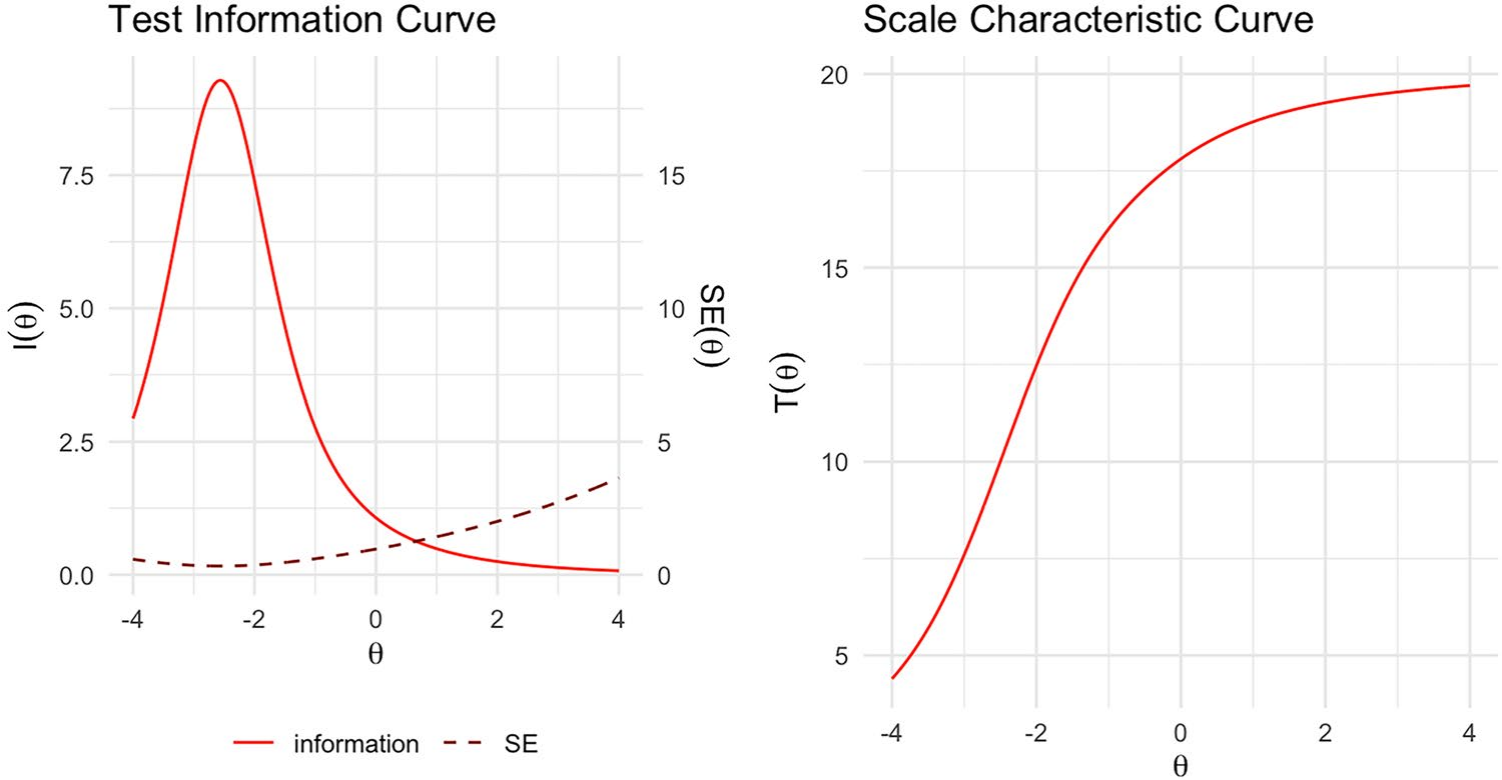
Multiscent-20 test information and scale characteristic curves. The left panel displays the item information curve (red line) and the standard error (dotted brown line). The right panel presents the scale characteristic curve. The test is most informative for a range of θ values between − 4 and − 1, indicating a higher precision and better discrimination in estimating the loss of odour identification ability for individuals within this ability range.

The test information curve (TIC; Fig. 3, left panel) illustrates the diagnostic test’s precision across the spectrum of the latent trait, reflecting its capacity to differentiate between individuals possessing varying levels of the trait. The scale characteristic curve (SCC; Fig. 3, right panel) also serves as an invaluable resource for evaluating diagnostic tests, encompassing facets such as test reliability, precision, measurement gap identification, and test calibration and equating. The SCC revealed that individuals with a θ value of approximately 0, corresponding to average latent ability, obtained an identification score of approximately 17–18 points. In contrast, those with a θ value of − 4 to − 2, indicating very low levels of odour identification ability, attained identification scores between 4 and 12.5.

### Influence of demographic factors

The one-way ANOVA revealed a statistically significant difference in the number of correct answers across the three educational attainment categories (F(2, 1829) = 13.55, p < 0.001). The Tukey honestly significant difference (HSD) test showed lower performance in the primary education group than in the secondary and tertiary education groups (primary vs. secondary education: mean difference = 1.05, 95% CI [0.53, 1.57], p < 0.001; primary vs. tertiary education: mean difference = 1.26, 95% CI [0.69, 1.83], p < 0.001). However, no statistically significant difference was found between the secondary education (high school) and tertiary education (college) groups (mean difference = 0.21, 95% CI [− 0.10, 0.52], p = 0.257).

A multiple linear regression analysis was conducted to examine the relationship between the Multiscent-20 identification score (the dependent variable) and sex, age group, and educational attainment (independent variables). The results indicated that the educational attainment was significantly associated with the Multiscent-20 identification score (secondary education: p = 6.94e−06, tertiary education: p = 6.26e−07). Specifically, participants with secondary and tertiary education had higher scores than those with primary education.

However, no significant relationship was found between age group and Multiscent-20 identification score (30–40 years: p = 0.97, 40–50 years: p = 0.89, 50–60 years: p = 0.17, > 60 years: p = 0.50). The analysis did not reveal any significant differences in olfactory performance between sexes with the Multiscent-20 identification score (p = 0.096). The multiple R^2^ value of 0.018 indicated that the model explained only a small proportion of the variation in the Multiscent-20 identification score.

### Descriptive data of the preliminary “normative” sample

A total of 533 individuals were excluded from the analysis of the preliminary normative curve. Exclusions were made according to the following criteria: recent infection (n = 240), illiteracy (n = 16), severe memory impairment (n = 34), prior head trauma (n = 107), nasal surgery (n = 101), persistent post-COVID-19 hyposmia (n = 4), self-reported hyposmia (n = 135), diagnosed neurological disease (n = 13), or scores of 23 or less on the Mini-Mental State Examination (MMSE) (n = 18).

**Figure 4:**
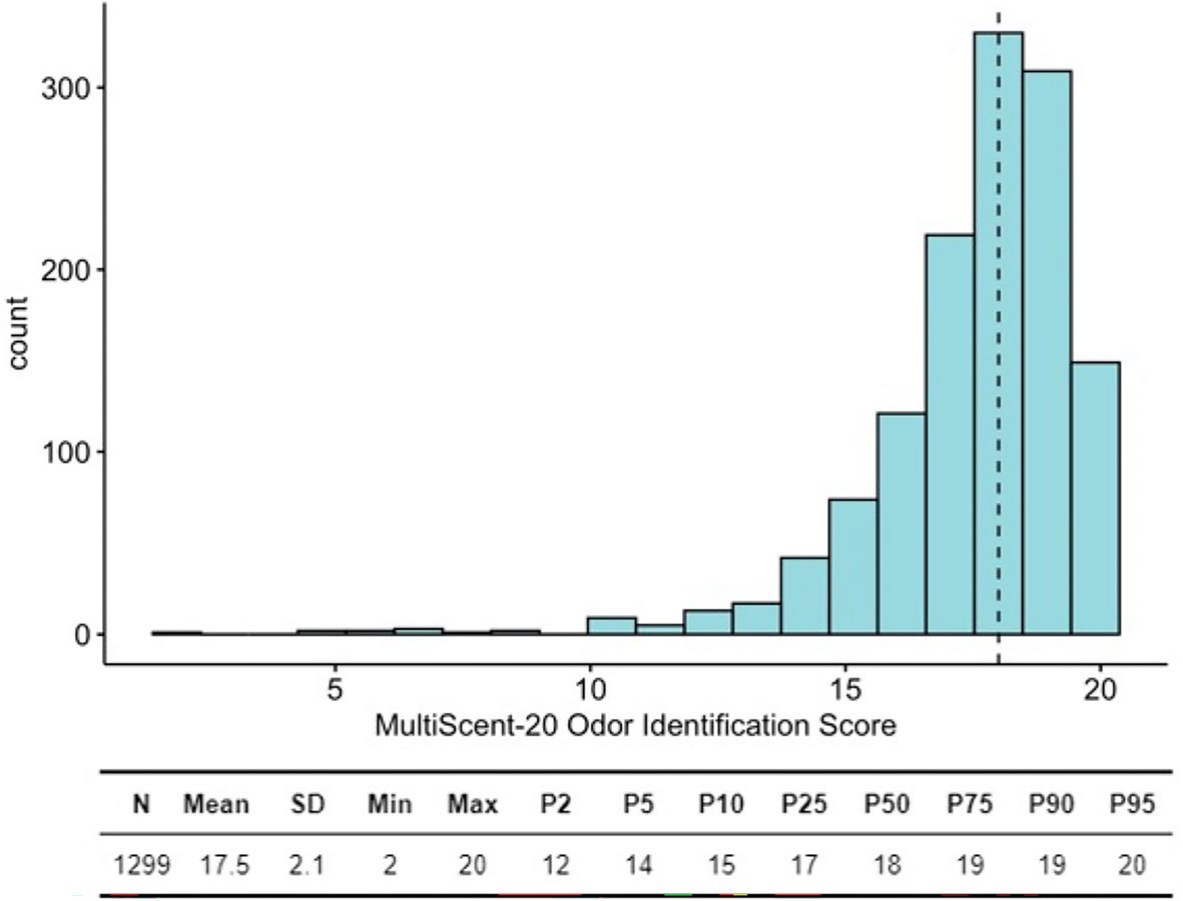
MultiScent-20 Odour Identification Score: distribution and descriptive statistics of central tendencies, dispersion, and percentiles of normative performance.

The sample of 1299 individuals had a mean Multiscent-20 identification score of 17.5, with a standard deviation of 2.1, as seen in Fig. 4. The median score was 18, and the trimmed mean was 17.8, indicating that the central tendency of the scores was relatively high. The scores ranged from a minimum of 2 to a maximum of 20. The skewness value of − 2.3 suggests a negative (left) skew, indicating that the majority of the participants scored higher on the Multiscent-20 identification scale, with a smaller number of individuals scoring lower. The kurtosis value of 7.3 implies a leptokurtic distribution, meaning that the scores were more concentrated around the mean than in a normal distribution.

### Assessment of internal and external validity, and test–retest reliability

#### Comparison of average performance: olfactory dysfunction vs. normosmic groups

**Figure 5:**
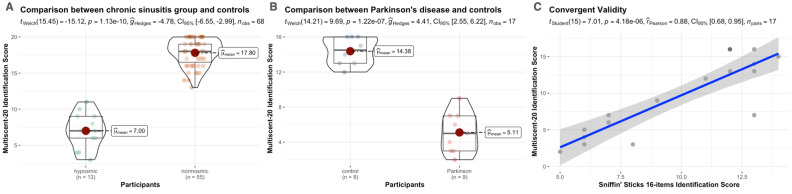
MultiScent-20 in Olfactory Function Assessment. (**A**) Compares MultiScent-20 scores between chronic sinusitis patients (hyposmic group) and normosmic controls, showing a significant difference. (**B**) Compares scores between Parkinson’s disease patients and age-matched controls, indicating a substantial olfactory deficit in Parkinson’s. (**C**) Scatter plot demonstrating the convergent validity of MultiScent-20 with the Sniffin’ Sticks 16-item score. Violin plots show score distributions; central red dot is the mean. Statistical details (effect sizes, p-values, CI 95%, n obs/n pairs) are provided.

Patients diagnosed with olfactory dysfunction resulting from chronic sinusitis demonstrated an average performance score of 7.0 ± 2.4. This score is significantly different from the normosmic control group’s average score of 17.8 ± 1.84 (Welch’s t-test, t(15.5) = 15.1, p < 0.001, mean difference = 10.8, Cohen’s d = 5.03, Levene’s F(1, 66) = 1.02, p = 0.317, Shapiro–Wilk p = 0.011; Anderson–Darling p = 0.003) (Fig. 5A).

Additionally, the subgroup comprising nine patients with Parkinson’s Disease, who are recognized to suffer from olfactory disorders^9^ recorded an average identification score of 5.11 ± 2.32. This was in contrast to age- and education-matched controls (n = 8), who achieved an average score of 14.38 ± 1.60 (Student’s t-test, t(15) = 9.472, p < 0.001, 95% CI [7.18, 11.35], Shapiro–Wilk p = 0.162, Levene’s F(1, 15) = 1.34, p = 0.265). The test revealed a highly significant mean difference with an extremely large effect size (Cohen’s d = 4.60). It is important to highlight that the control group, being older, exhibited a lower performance relative to the normative sample (Fig. 5B).

#### Test–retest reliability

The evaluation of test–retest reliability for the olfactory test, conducted over an average interval of 31.73 days (SD = 2.1 days), revealed a Pearson’s r correlation coefficient of 0.94 (95% CI [0.91–0.96]). A linear model demonstrated a strong predictive capability for ‘retest’ scores based on ‘test’ scores, with a significant positive correlation (std. ꞵ = 0.94) and explaining 89% of the variance. The model’s intercept was statistically significant at 1.60. Additionally, the Intraclass Correlation Coefficient (ICC) for a one-way random model, measuring agreement (ICC1), was 0.93 with a 95% confidence interval ranging from 0.89 to 0.95. The tenfold cross-validation results also indicate high reliability in test–retest measurements, with a mean ICC of 0.9218. The median ICC was 0.9579 (range 0.809–0.987), demonstrating high reliability even in the worst cases. This result indicates a strong consistency in the test outcomes over time among the participants.

#### Correlation and agreement with Sniffin’ Sticks test (SS-16)

Pearson’s correlation analysis conducted to assess the convergent validity between the MultiScent test and the Sniffin’ Sticks test (SS-16), a well-known olfactory test, yielded significant results among both Parkinson’s Disease (PD) patients and corresponding control group. With a correlation coefficient (r) of 0.88 and a 95% confidence interval ranging from 0.68 to 0.95 (p < 0.001), the analysis demonstrated a strong positive relationship between the two olfactory assessments. Furthermore, the determination coefficient (R^2^) of 0.77 indicates that approximately 77% of the variance in olfactory test scores between the SS-16 and MultiScent-20 tests is shared, underscoring the substantial overlap in the measures of olfactory function provided by both tests (Fig. 5C).

#### Internal consistency

McDonald’s omega reliability index was determined to be 0.902, indicating high internal consistency. The 95% confidence interval for this measure ranged from 0.888 to 0.916.

## Discussion

In the present study, we developed a novel digital odour identification test presented on a dedicated tablet that releases odours and evaluated its psychometric properties. This new test is automated and portable, enabling standardized olfactory stimuli delivery, swift recording, and scoring of responses in a cost-effective manner. The validity of the test was evaluated with the IRT framework. The findings revealed that the 2PL model yielded the most appropriate fit to the data, indicating variations in item discrimination and difficulty. Furthermore, the adequate fit (based on infit and outfit statistics) showed that the majority of participant responses were in line with the model expectations.

The TIC and SCC provide insights into the performance of diagnostic tests in general and may also be used for odour identification tests such as in the present study. The TIC provides a visual representation of the test’s capability to differentiate between individuals with different ability levels. For the MultiScent-20, the TIC revealed that the test has a high level of precision across lower levels of the latent trait, suggesting that it can effectively differentiate between individuals with hyposmia and anosmia. The SCC showed that individuals with an average odour identification ability obtained a score of 17–18 points, indicating that they correctly identified most of the scents presented. In contrast, individuals with the lowest levels of olfactory ability (θ values lower than − 2) obtained lower scores, ranging between 4 and 12.5 points.

The present findings also highlighted variations across demographic groups, consistent with the complexity of olfactory function testing. The data showed that while higher education levels were correlated with better performance on the Multiscent-20, sex was only marginally associated with olfactory ability. This is in line with previous research finding sex differences in olfaction with relatively small effect sizes^10^. Moreover, previous research has shown that in odour identification, verbal ability^11^, educational attainment^12^, and cognitive factors^13,14^ play a significant role in modulating both odor discrimination and identification capabilities.

The percentile-based interpretation of the normative values was guided by the contribution of the ‘Sniffin’ Sticks’ test and the UPSIT, prompting us to consider a score below the 10th percentile as indicative of hyposmia^1,4^. A similar standardized classification system for an objective assessment of participant performance based on percentiles is included in the guidelines of the American Academy of Clinical Neuropsychology consensus conference statement on labelling performance in tests with non-normal distributions^15^. In this classification, scores above the 24th percentile (≥ 17 points) are classified “Within Normal Expectations Score”, “Within Normal Limits Score”, or “normosmia”, while scores ranging from the 10th to the 24th percentile (15–16 points) are labelled as “Low Average Score” but still normosmia. Scores falling between the 2nd and 8th percentiles are designated as “Below Average Score” or “hyposmia”, while scores below the 2nd percentile are classified as “Exceptionally Low Score” or “anosmia”. Following these guidelines provided a consistent and objective assessment of subject performance in our study.

The significant difference in performance scores between patients with olfactory dysfunction due to chronic sinusitis and the normosmic control group is a critical finding. With a marked mean difference and a large effect size (Cohen’s d = 5.03), the study demonstrates the extent of olfactory impairment in individuals with chronic sinusitis. These results are consistent with the existing literature that documents the impact of chronic sinusitis on olfactory capabilities. Moreover, the subgroup of PD patients showed a more pronounced olfactory deficit compared to their matched controls. The substantial effect size (Cohen’s d = 4.60) in this comparison highlights the severity of olfactory impairment in PD, aligning with prior research indicating olfactory dysfunction as a common non-motor symptom of PD. This stark contrast between PD patients and controls further validates the sensitivity of the olfactory test in detecting olfactory deficits in neurodegenerative conditions.

The high test–retest reliability observed in this study is indicative of the consistency and stability of the olfactory test over time. This finding is particularly relevant for clinical settings where repeated measurements are essential to monitor the progression of olfactory dysfunction or the efficacy of therapeutic interventions. The strong correlation coefficient suggests that the test can reliably measure olfactory function over a period of about one month, an important consideration for longitudinal studies.

The strong positive correlation between the MultiScent-20 test and the Sniffin’ Sticks test (SS-16) underscores the convergent validity of these olfactory assessments. The high correlation coefficient (r = 0.88) and determination coefficient (R^2^ = 0.77) indicate that both tests are measuring similar constructs of olfactory function.

The olfactory function test presented with this digital device can enable large-scale administration without the need for examiners, as scoring is automatic, and the test is self-administered. This feature facilitates epidemiological screening in populations^16–18^. Moreover, the equipment enables the collection of multidimensional data, including discrimination, threshold, familiarity, pleasantness, and preference for different odours. The collection and analysis of multidimensional data provide a more comprehensive and detailed understanding of olfactory responses and individual or collective patterns of odour perception^19^. Furthermore, the device facilitates the utilization of artificial intelligence, as analysis of the generated big data can provide novel insights into the biology of olfactory perception and the cognitive processes involved in odour identification^13^. The application of IRT may also be of interest in enabling computerized adaptive tests.

Moreover, incorporating “universal odours” in the Multiscent-20 and the initial exploration of cross-cultural research underlines the tool’s potential to transcend cultural boundaries^20,21^. Subsequent studies may refine the test by addressing any discrepancies in item calibration and investigating other potential factors that could account for variation in test performance.

The development of the Multiscent-20 marks a significant advancement in olfactory testing, shifting from traditional, manual methods like the UPSIT and Sniffin’ Sticks to a digital approach that enhances scalability and reduces the need for trained personnel^1,3^. This device automates odor delivery and response scoring, integrating algorithms for precise analysis. The use of a digital platform allows for large-scale screenings and complex data analysis, significantly improving upon the limitations of traditional methods and setting a new standard in olfactory research^22^.

This study also has some limitations. It is crucial to acknowledge that the multiple linear regression model explained only a small proportion of variance in Multiscent-20 identification scores, emphasizing the need for future research to identify additional factors influencing olfactory ability. The study’s participant sample, primarily comprising workers from a glass factory, may not fully represent the broader population, particularly of ethnic, cultural diversity, socioeconomic status, and educational background. The proposed cut-off values are preliminary and based on the recommendation by Guilmette et al.^15^. These values are subject to updates with the expansion of the normative sample, as well as the inclusion of more patients with olfactory dysfunction of known aetiology.

These limitations highlight the importance of expanding future research to diverse demographics to enhance the generalizability of findings. Moreover, it underscores the importance of refining Multiscent-20 test items with misfits, suggesting future modifications in odour concentration and test design to improve clinical relevance while acknowledging the test’s ongoing evolution.

In conclusion, the findings of the present study support the validity of the Multiscent-20 as an odour identification test. The 2PL IRT model provides a robust framework for understanding the performance on test items. The test represents an important step forward in olfactory function testing, with potential benefits for both clinical practice and research.

## Methods

### Study design and ethical approval

The study was approved by the Ethics Committee of the Faculty of Medicine of the University of Brasilia (Approval Number: 43188021-9-1001-5558). All methods were performed in accordance with the guidelines and regulations of the National Council of Research Ethics and the Declaration of Helsinki, and informed consent was obtained from all participants. Participant data are protected by national data protection legislation. The study had a cross-sectional observational design and utilized nonprobabilistic convenience sampling by quotas. In the following sections, we provide a detailed description of the experimental methodology and preliminary results.

### Participant enrolment and inclusion criteria

To be eligible for participation, individuals had to be at least 18 years of age and native speakers of Portuguese. The study participants consisted of healthcare professionals from the university hospital, medical students, administrative staff, and employees from a glass factory. We excluded participants who provided careless or potentially biased responses to the olfactory function test, such as selecting answers at random or choosing the same option for all responses. We purposely did not exclude individuals who had a recent cold or flu or who reported olfactory loss because a sample with sufficient variability in test performance is essential to evaluate the test’s ability to measure the loss of the sense of smell.

However, for the normative curve of the test, other inclusion and exclusion criteria were established to ensure the inclusion of normosmic individuals. Participants had to be 18 or older, native speakers of Portuguese, and free of any known impairment of smell or taste. Participants who had been sick (with a cold or flu) within 15 days prior to the exam were excluded, as were those who reported neurological or psychiatric diseases and those with a history of head trauma. Participants who demonstrated negligent performance in the exam were also excluded. Additionally, those aged above 60 years and scoring below 24 points on the MMSE were excluded^23^.

### Description of device features

**Figure 6:**
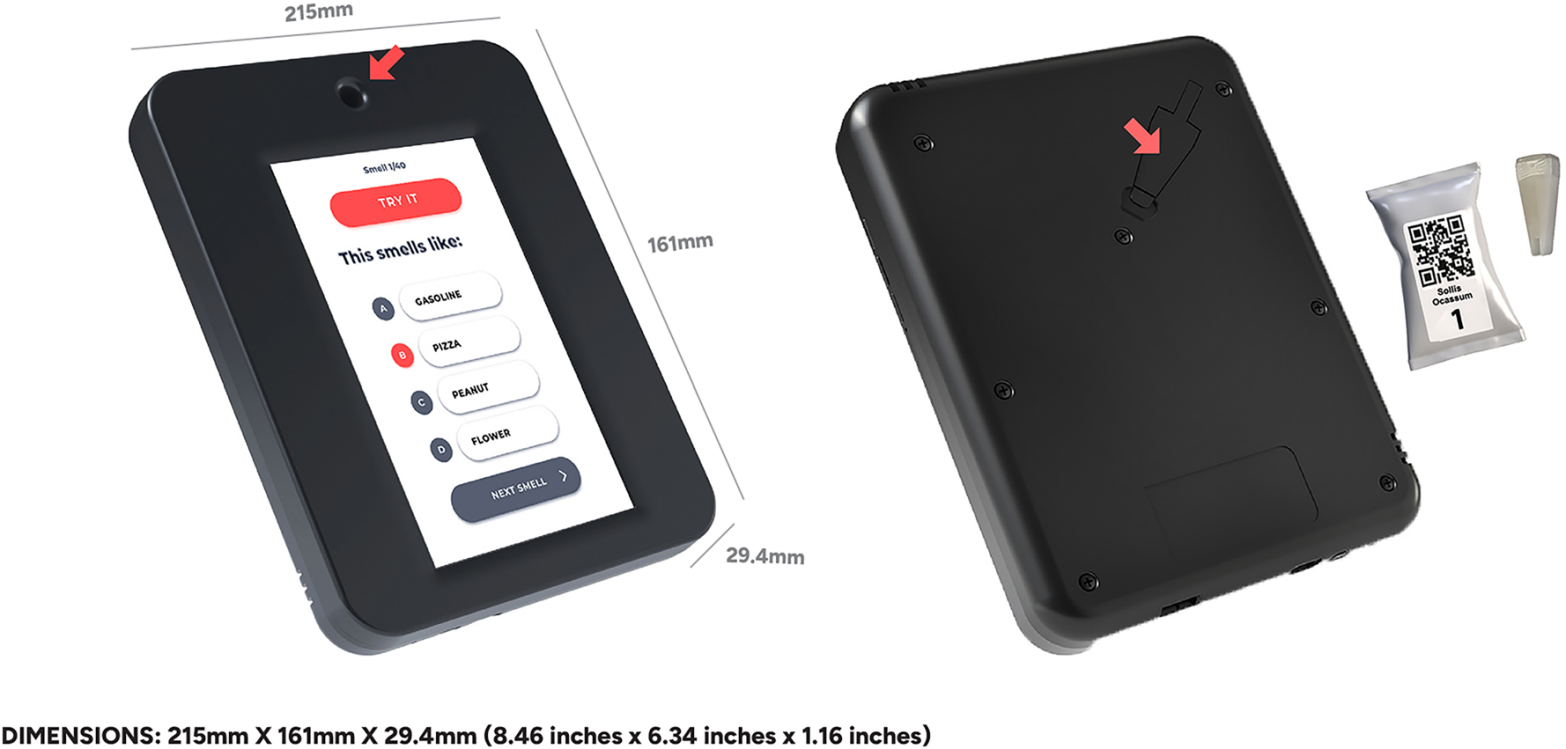
Front (left) and back (right) views of the Multiscent-20. It is a dedicated tablet with a 7-inch touchscreen used to present aromas and record responses digitally. The opening for odour release is indicated by an arrow on the front of the device. The fragrance capsule is also shown. The device can be loaded with up to 20 capsules made of oil-resistant polymer, in which olfactory stimuli are stored. The capsules are loaded through an insertion port (arrow) on the back of the device.

The Multiscent-20 is a dedicated tablet featuring a 7-inch touchscreen specifically designed for use in the cosmetics industry to present perfume fragrances to customers (Fig. 6)^5^.

The Multiscent-20 is a portable tablet computer with an integrated hardware system consisting of a central processing unit, touchscreen, Wi-Fi antenna, USB dock, power supply, and rechargeable battery. Additionally, it contains an odour system composed of 20 microcartridges, an air filter, a system that generates a dry air stream, and an odour-dispensing opening. The device is capable of presenting 20 different odours from individual odour capsules, which store the olfactory stimuli by incorporating the odours into an oil-resistant polymer. The capsules are loaded through an insertion port on the back of the device.

A software-controlled processing unit governs the flow of dry air, generating a constant air stream that passes through the capsule, releasing individual odours through a small opening at the upper front of the device. In this study, the intra-stimulus interval, i.e., the release of each odour stimulus, had a duration of 5 s, and odour release was initiated by participant activation via a touch screen interface. The participants had complete autonomy to activate the next odour at their own pace. The minimum inter-stimulus interval is 6 s, but the actual interval tended to be longer, as participants are required to read four descriptors before releasing each odour. Each capsule contained 35 µL of oil-based odour solution, allowing the device to maintain consistent odour intensity and identifiability for up to 100 activations, as per the manufacturer’s instructions.

A digital application (software) was developed to present odours and record olfactory function test results. The device delivers odours through a dry air system, leaving no residue in the environment or on users. Users can access the test using an application on the device (screen version) or mobile phone. The results are accessible through the integrated software, allowing for storage and analysis of the data.

### Selection of the “universal” odours

The methodology employed for selecting “universal” odours for the Multiscent20 olfactory assessment tool was anchored in three pivotal criteria, each odour being required to meet at least two of these conditions for its inclusion. Primarily, an odour had to be included within the scope of scents identified in the study conducted by Schriever et al.^6^. Initially designed for a paediatric cohort with a selection of 12 items, our research adapted this approach to serve the adult population, positing that odours recognizable by children would be even more identifiable to adults, attributed to their broader spectrum of life experiences and exposures.

Secondly, among the 77 odours used in major olfactory tests described in the literature, (Supplementary Table S3) we identified 35 odours that were utilized in two or more tests. Therefore, the second criterion necessitated that an odour be among these 35 of the 77 odours mentioned in the literature.

Thirdly, the odours chosen were required to have demonstrated a minimum identification accuracy of 80% or higher in a proof-of-concept study by Nakanishi et al.^5^, ensuring their high likelihood of accurate recognition. Additionally, factors including worldwide prevalence and the commercial availability across a global scale, covering five continents, were also taken into account.

The methodology employed for the selection of distractors was adapted from the framework established by Schriever et al.^6^: 1. *Four-Alternative Forced-Choice (4-AFC) Procedure* The 4-AFC format was implemented, presenting participants with each odor accompanied by one correct answer and three distractors. 2. *Target-Related Selection* In the distractor selection process, one of the three distractors for each odor was chosen for its semantic relation to the target odor, thereby enhancing the test’s discriminatory demand. This inclusion of semantically related and unrelated distractors aimed to increase the identification task’s complexity, necessitating more refined differentiation skills from the participants. 3. *Odor Selection Variability* The selection of odors was diversified across various levels of difficulty to present a graduated challenge to all participants. This variation was intended to prevent the “ceiling effect”, where the simplicity of the test might lead to disproportionately high scores across participants, thus undermining the test’s discriminative capacity. (Supplementary Table S1).

The selected odours were Smoked, Lavender, Coconut, Mint, Vanilla, Tire, Rose, Coffee, Grass, Bubble-gum, Menthol, Grape, Clove, Strawberry, Banana, Orange, Cinnamon, Garlic, Pizza, and Onion. Specifications are delineated in Supplementary Table S4.

### Experimental setup

The Multiscent-20 odour identification test commences with the participant reviewing the step-by-step instructions displayed on the initial screen. These instructions are presented below:

1. This assessment consists of a test containing 20 distinct odours.
2. Enter your identification information and press the “next” button.
3. The next screen will show a “Try it” button, the phrase “This smell resembles”, and with four alternative answers. Please reading all the choices before pressing the “Try it” button.
4. Upon pressing the “Try it” button, a small opening at the top front of the device will release the odour for 5 s. You can press the “Try it” button up to three times. Please maintain the device approximately 10 cm from your nostrils after pressing the button. After perceiving the odour, please select one of the alternatives, touch the corresponding letter, and press the “next odour” button to proceed.
5. The number of correct responses will be displayed upon completion of the test.

This assessment was a 4-AFC test, meaning that participants were asked to choose one of the four alternatives to advance to the next odour. The software was specifically developed to present questions and manage responses on the device. After the test, the results are displayed, and the device automatically synchronizes with the database stored in the cloud.

### Data analysis (descriptive statistics and dimensionality assessment)

For the descriptive analysis, categorical variables are presented as the frequency (n) and percentage (%). Continuous variables are either described as the mean and standard deviation (SD) or median and interquartile range (IQR), depending on the normality of the data, as determined with the Shapiro‒Wilk test. Data analysis was mainly performed in R software (v4.3.0, R Foundation for Statistical Computing, Vienna, Austria). All statistical tests were two-sided, with a significance threshold set at 0.05.

To assess the dimensionality of the dataset, a CFA was performed using JASP^24^. Model fit indices, such as the SRMSR, TLI, and RMSEA, were examined to evaluate the model fit. This procedure aimed to evaluate the suitability of the dataset for further analysis using an IRT model.

### Item response theory analysis

To conduct the IRT analysis, the ‘mirt’ package^25^ in R was utilized. The following steps were followed for the analysis:

- *Data preparation* Data were organized into a matrix format, with rows representing individual participants, and columns representing dichotomous responses (correct/incorrect) to each of the 20 odour items.
- *Model estimation* Using the ‘mirt’ function, the parameters of the 1PL, 2PL, and 3PL models were estimated. The 1PL model assumed a constant discrimination parameter across items, while the 2PL model allowed for variation in discrimination parameters. The 3PL model additionally included a guessing parameter, accounting for the possibility that participants may have guessed the correct answer by chance.
- *Model comparison* To determine the best-fitting model for the present dataset, goodness-of-fit statistics were compared, such as the AIC and BIC. Lower values of these statistics indicated a better-fitting model.
- *Item analysis* For the best-fitting model, the ICCs were examined. These provide information about the relationship between participant latent trait levels and their probability of correctly identifying each odour. Item information functions (IIFs) were examined to understand the precision of each item in measuring the latent trait.

### Influence of demographic factors (sex, age, and educational attainment)

To investigate the relationship between participant educational attainment and the number of correct answers on the administered test, a one-way ANOVA was conducted. Participants were divided into three categories based on their educational attainment: primary (the fundamental level in Brazil), secondary (high school), and tertiary (college) education. The dependent variable was the number of correct answers in the odour identification test. Subsequently, Tukey’s HSD test was employed to identify pairwise differences between the three educational attainment groups, provided that the ANOVA yielded significant results.

A multiple linear regression model was employed to investigate the relationship between the Multiscent-20 identification scores (dependent variable) and sex, age group, and educational attainment (independent variables). The dataset was analysed using R software, with the “lm” function utilized to create the regression model.

### Evaluation of internal and external validity, and assessment of test–retest reliability

#### Subjects

- Administrative staff were selected from the sample described in “Participants” section, comprising 55 healthy individuals without olfactory complaints, including 25 men and 30 women (mean age = 31.7 years, SD = 4.3 years, range 21 to 42 years). Additionally, 13 individuals, comprising 5 men and 8 women (mean age = 57.4 years, SD = 16.8 years, range 24 to 75 years), diagnosed with hyposmia or anosmia and chronic sinusitis^26^ who were receiving follow-up care at the otorhinolaryngology outpatient clinic of the University Hospital of Brasília.
- Parkinson’s Disease Subgroup and Matched Control. Nine patients diagnosed with Parkinson’s Disease^27^ (mean age = 65.8 years, SD = 11.5 years, range 47 to 78 years), followed in the neurology outpatient clinic, and eight age- and education-matched control subjects (mean age = 61.4 years, SD = 6.2 years, range 53 to 71 years) were selected for the comparison of olfactory performance and cross-validation study with the Sniffin’ Sticks test. This difference in age was not statistically significant (p = 0.352). Gender distribution across the groups was not significantly different (p = 0.772), with 62.5% females (N = 5) in the control group and 55.6% females (N = 5) in the Parkinson’s group. Males constituted 37.5% (N = 3) of the control group and 44.4% (N = 4) of the Parkinson’s group.

#### Comparison of average performance: olfactory dysfunction vs. normosmic groups

With the aim of evaluating the differences in olfactory performance among three groups—those with olfactory dysfunction due to chronic sinusitis, a normosmic control group, and the Parkinson’s Disease Subgroup and Matched Control—we employed t-tests.

#### Test–retest reliability

To evaluate the temporal stability of olfactory test outcomes, we conducted the assessment on the same cohort of individuals at two distinct time points, separated by an average interval of 31.7 days (sd = 2.1). For the analysis, Pearson’s correlation coefficient and the Intraclass Correlation Coefficient (ICC) were employed to quantify the degree of consistency between the two sets of measurements. We implemented a tenfold cross-validation approach using the entire test–retest dataset without partitioning it into groups. Using the ‘*caret’* package in R, we configured a tenfold cross-validation procedure, with each fold serving as a validation set once and the remaining folds as the training set. ICCs were calculated for each fold using the ‘*icc’* function from the ‘*irr’* package. This approach enhances generalizability by ensuring that the findings are not contingent upon a specific data subset and mitigating overfitting. Furthermore, a linear regression model was applied, designating the results of the retest as the dependent variable and the initial test outcomes as the independent variable, to investigate the predictive relationship between the two measurements.

#### Correlation with Sniffin’ Sticks test (SS-16)

To determine the convergent validity with a well-known test that measures the same construct, a correlation analysis was conducted to examine the relationship between the Sniffin’ Sticks (SS-16) and MultiScent-20 test scores. The Pearson correlation coefficient (r) was calculated along with its corresponding p-value, and the 95% confidence interval (CI) limits.

#### Internal consistency

From a Confirmatory Factor Analysis (CFA) conducted using Mplus software (version 8.8), we modelled all items as indicators of a single latent factor, with factor loadings, thresholds, and variances extracted to determine model fit, using indices such as RMSEA, CFI, TLI, and SRMR. Subsequently, we used the NumPy library (in python), to calculate McDonald’s omega reliability coefficient^28^. This step involved extracting standardized factor loadings from the Mplus output and computing error variances for each item.

This coefficient, representing the scale’s internal consistency, is crucial for measuring the reliability of the scale in measuring the intended construct.

### Supplementary Information

Supplementary Tables.

## Data Availability

The anonymized data that support the findings of this study are openly available in Zenodo public repository, at 10.5281/zenodo.8079860 reference number 8079860.
