# Supplementary material for: Development and validation of the MultiScent-20 digital odour identification test using item response theory
Source: Sci Rep. 2024 Jul 1;14:15059. doi: 10.1038/s41598-024-65915-3 (PMC11219931; doi:10.1038/s41598-024-65915-3)
Supplement: Supplementary file 1 — Supplementary Tables. [file 41598_2024_65915_MOESM1_ESM.docx]

**Supplementary materials**

**Title: Development and Validation of the MultiScent-20 Digital Odour Identification Test Using Item Response Theory**

Authors: Marcio Nakanishi^1,^ *, Pedro Renato de Paula Brandão^2,^ *, Gustavo Subtil Magalhães Freire^1^, Luis Gustavo do Amaral Vinha^3^, Marco Aurélio Fornazieri^4^, Wilma Terezinha Anselmo Lima^5^, Danilo Assis Pereira^6^, Gustavo Henrique Campos de Sousa^2^, Claudia Galvão^7^, Thomas Hummel^8^

**Supplementary materials**

**Supplementary Table S1.** Items of the Multiscent-20 odour identification test.

| **Number** | **Odourants (Target)** | **Distractors** |
| --- | --- | --- |
| 1 | **Smoke** | coconut, mint, **smoke**, cherry |
| 2 | **Lavender** | caramel, **lavender**, rosemary, popcorn |
| 3 | **Coconut** | watermelon, clove, **coconut**, gasoline |
| 4 | **Mint** | **mint**, coffee, garlic, banana |
| 5 | **Vanilla** | onion, **vanilla**, pizza, fish |
| 6 | **Tire** | orange, mint, apple, **tire** |
| 7 | **Rose** | **rose**, caramel, garlic, eucalyptus |
| 8 | **Coffee** | pizza, grape, lavender, **coffee** |
| 9 | **Grass** | chocolate, **grass**, pineapple, rose |
| 10 | **Gum** | **gum**, clove, butter, gasoline |
| 11 | **Menthol** | honey, peanut, **menthol**, watermelon |
| 12 | **Grape** | bacon, **grape**, flower, mint |
| 13 | **Clove** | lemon, wood, **clove**, rose |
| 14 | **Strawberry** | **strawberry**, onion, grass, smoke |
| 15 | **Banana** | popcorn, coffee, orange, **banana** |
| 16 | **Orange** | caramel, **orange**, vanilla, eucalyptus |
| 17 | **Cinnamon** | **cinnamon**, smoke, mint, peanut |
| 18 | **Garlic** | cherry, apple, **garlic**, rosemary |
| 19 | **Pizza** | **pizza**, mint, strawberry, soda |
| 20 | **Onion** | chocolate, coconut, lavender, **onion** |

Legend. The table shows the 20 odours, their descriptors, and distractors.

Target odours are in bold.

**Supplementary Table S2.** Discrimination and Difficulty Parameters of the Multiscent-20 Odour Identification Test.

| Item | Discrimination (a) | Difficulty (b) |
| --- | --- | --- |
| Smoke | 1.201 | -1.876 |
| Lavender | 0.790 | -2.039 |
| Coconut | 0.960 | -2.587 |
| Mint | 1.934 | -2.345 |
| Vanilla | 2.427 | -2.741 |
| Tire | 0.754 | -2.169 |
| Rose | 0.378 | -0.599 |
| Coffee | 0.555 | -3.219 |
| Grass | 0.585 | -1.737 |
| Bubblegum | 1.705 | -2.736 |
| Menthol | 2.417 | -2.474 |
| Grape | 0.870 | -2.435 |
| Clove | 1.038 | -2.266 |
| Strawberry | 1.892 | -2.310 |
| Banana | 1.139 | -3.026 |
| Orange | 0.791 | -2.096 |
| Cinnamon | 0.551 | -0.974 |
| Garlic | 1.630 | -2.940 |
| Pizza | 1.505 | -2.612 |
| Onion | 1.823 | -2.599 |

Legend: This table shows the discrimination and difficulty parameters for each of the 20 odours in the Multiscent-20 odour identification test.

**Supplementary Table S3.** a) Olfactory tests and number of odours

| **Name of the Test (author, year)** | **Number of odours** |
| --- | --- |
| 1. Connecticut Chemosensory Clinical Research Center Test   (Cain et al., 1983) | 8 |
| 1. University of Pennsylvania Smell Identification Test   (Doty, 1984) | 40 |
| 1. T&T Olfactometer Test (Takagi, 1989) | 5 |
| 1. Sniffin' Sticks Test (Hummel et al., 1996) | 16 |
| 1. Cross-Cultural Smell Identification Test   (Doty et al., 1996) | 12 |
| 1. Smell Diskettes   (Briner and Simmen,1999) | 8 |
| 1. Barcelona Smell Test - 24   (Cardesín et al., 2006) | 24 |
| 1. The 40-item Monell Extended Sniffin' Sticks Identification Test -MONEX-40 (Freiher et al., 2011) | 40 |
| 1. Pediatric Smell Wheel (Cameron & Doty, 2013) | 8 |
| 1. International Odor Identification Test for Children   (Schriever VA, 2018) | 8 |

References: 1. Cain, W. S., Gent, J., Catalanotto, F. A. & Goodspeed, R. B. Clinical evaluation of olfaction. Am J Otolaryngol 4, 252–256 (1983). 2.Doty, R. L. et al. Development of the University of Pennsylvania Smell Identification Test: A Standardized Microencapsulated Test of Olfactory Function. Physiol Behav 32, 489–502 (1984). 3.Takagi, S. University of Tokyo. in Olfaction 481 (Tokyo, 1985). 4.Hummel, T., Sekinger, B., Wolf, S. R., Pauli, E. & Kobal, G. ‘Sniffin’ Sticks’: Olfactory Performance Assessed by the Combined Testing of Odor Identification, Odor Discrimination and Olfactory Threshold. Chem Senses 22, 39–52 (1997). 5.Doty, R., Marcus, A. & Lee, W. Development of the 12-Item Cross-Cultural Smell Identification Test (CC-SIT). Laryngoscope 3, 353–6 (1996). 6.Briner, H. R., Simmen, D. & others. Smell diskettes as screening test of olfaction. Rhinology 37, 145–148 (1999). 7.Cardesin, A. et al. Barcelona Smell Test-24 (BAST-24): validation and smell characteristics in the healthy Spanish population. Rhinology 44, 83 (2006). 8.Freiherr, J. et al. The 40-item monell extended sniffin’sticks identification test (MONEX-40). J Neurosci Methods 205, 10–16 (2012). 9.Cameron, E. L. & Doty, R. L. Odor identification testing in children and young adults using the smell wheel. Int J Pediatr Otorhinolaryngol 77, 346–350 (2013). 10.Schriever, V. A. et al. Development of an International Odor Identification Test for Children: The Universal Sniff Test. Journal of Pediatrics 198, 265-272.e3 (2018).

**Supplementary Table S3.** b) Odours recurring and their counts from the main tests described in item a.

| **Odour** | **Repetitions** |
| --- | --- |
| Banana | 7 |
| Rose | 7 |
| Lemon | 6 |
| Cinnamon | 6 |
| Pineapple | 5 |
| Onion | 5 |
| Chocolate | 5 |
| Peach | 4 |
| Coffee | 4 |
| Soap | 4 |
| Turpentine | 4 |
| Orange | 4 |
| Smoke | 3 |
| Coconut | 3 |
| Cherry | 3 |
| Garlic | 3 |
| Gasoline | 3 |
| Grass | 3 |
| Leather | 3 |
| Strawberry | 3 |
| Liquorice | 3 |
| Peanut | 3 |
| Vanilla | 2 |

**Supplementary Table S4**. Specifications of odorant compounds used in the Multiscent20 olfactory assessment test: undiluted oil-based odour solutions.

| Position | Odorant | Code | Supplier |
| --- | --- | --- | --- |
| 1 | Smoke | HU45100112791 | Givaudan |
| 2 | Lavender | 44523 | Solutaste |
| 3 | Coconut | FAV333934 | Fav105 |
| 4 | Mint | 90000424 | Fracarolli |
| 5 | Vanilla | HU45100110837 | Givaudan |
| 6 | Tire | EAM23630/00 | Givaudan |
| 7 | Rose | HU20625528546 | Givaudan |
| 8 | Coffee | EAM23942/00 | Givaudan |
| 9 | Grass | EAM23624/00 | Givaudan |
| 10 | Gum | HU20627102236 | Givaudan |
| 11 | Menthol | R0317 | Dierberger |
| 12 | Grape | EAM23629/00 | Givaudan |
| 13 | Clove | R0292 | Dierberger |
| 14 | Strawberry | EAM23945/00 | Givaudan |
| 15 | Banana | HU20625528555 | Givaudan |
| 16 | Orange | EAM23626/00 | Givaudan |
| 17 | Cinnamon | EAM23628/00 | Givaudan |
| 18 | Garlic | HU45100114483 | Givaudan |
| 19 | Pizza | 28531 | Solutaste |
| 20 | Onion | 44512 | Solutaste |
